# Nuclear magnetic resonance in conjunction with functional genomics suggests mitochondrial dysfunction in a murine model of cancer cachexia

**DOI:** 10.3892/ijmm.2010.557

**Published:** 2010-11-10

**Authors:** CATERINA CONSTANTINOU, CIBELY CRISTINE FONTES DE OLIVEIRA, DIONYSSIOS MINTZOPOULOS, SILVIA BUSQUETS, JIANXIN HE, MEENU KESARWANI, MICHAEL MINDRINOS, LAURENCE G. RAHME, JOSEP M. ARGILÉS, A. ARIA TZIKA

**Affiliations:** 1 NMR Surgical Laboratory, Massachusetts General and Shriners Hospitals, Harvard Medical School, Boston, MA 02114;; 2 Molecular Surgery Laboratory, Massachusetts General and Shriners Hospitals, Harvard Medical School, Boston, MA 02114, USA;; 3 Cancer Research Group, Departament de Bioquímica i Biologia Molecular, Facultat de Biologia, Universitat de Barcelona, Diagonal 645, Barcelona 08028, Spain;; 4 Athinoula A. Martinos Center of Biomedical Imaging, Department of Radiology, Massachusetts General Hospital, Boston, MA 02114;; 5 Department of Biochemistry, Stanford University School of Medicine, Stanford, CA 94305, USA

**Keywords:** skeletal muscle, cancer cachexia, mitochondria, mitochondrial, PGC-1ß, UCP3, FoXO3α, microarrays, genomics

## Abstract

Cancer patients commonly suffer from cachexia, a syndrome in which tumors induce metabolic changes in the host that lead to massive loss in skeletal muscle mass. Using a preclinical mouse model of cancer cachexia, we tested the hypothesis that tumor inoculation causes a reduction in ATP synthesis and genome-wide aberrant expression in skeletal muscle. Mice implanted with Lewis lung carcinomas were examined by *in vivo* ^31^P nuclear magnetic resonance (NMR). We examined ATP synthesis rate and the expression of genes that play key-regulatory roles in skeletal muscle metabolism. Our *in vivo* NMR results showed reduced ATP synthesis rate in tumor-bearing (TB) mice relative to control (C) mice, and were cross-validated with whole genome transcriptome data showing atypical expression levels of skeletal muscle regulatory genes such as peroxisomal proliferator activator receptor γ coactivator 1 ß (PGC-1ß), a major regulator of mitochondrial biogenesis and, mitochondrial uncoupling protein 3 (UCP3). Aberrant pattern of gene expression was also associated with genes involved in inflammation and immune response, protein and lipid catabolism, mitochondrial biogenesis and uncoupling, and inadequate oxidative stress defenses, and these effects led to cachexia. Our findings suggest that reduced ATP synthesis is linked to mitochondrial dysfunction, ultimately leading to skeletal muscle wasting and thus advance our understanding of skeletal muscle dysfunction suffered by cancer patients. This study represents a new line of research that can support the development of novel therapeutics in the molecular medicine of skeletal muscle wasting. Such therapeutics would have wide-spread applications not only for cancer patients, but also for many individuals suffering from other chronic or endstage diseases that exhibit muscle wasting, a condition for which only marginally effective treatments are currently available.

## Introduction

Cachexia is a complex metabolic syndrome that can result from adaptation to an underlying illness; it is characterized by loss of muscle mass with or without loss of fat mass (1). Selective targeting of skeletal muscle is a principal feature of cachexia pathophysiology (2), and a major cause of fatigue (3) in patients. Indeed, the condition can rob patients of 30% or more of their body weight (4). As many as half of untreated cancer patients present with cachexia (5,6), which is most commonly associated with cancers of the gastrointestinal tract and lung (6); furthermore, in these cancer types muscle wasting occurs at a faster rate than any other known situation in human subjects (6–8).

Cancer-induced muscle wasting is typically associated with the development of significantly increased resting energy expenditure (REE) in skeletal muscle and fat (6,7,9)-REE increases as a result of decreased caloric intake and/or increased energy expenditure. Uncoupling proteins (UCPs) have been implicated in the control of energy metabolism (6,7). They transport protons into the mitochondrial matrix (10) and nonesterified fatty acid (FA) anions out of the matrix in a process called FA cycling (11). Both of these processes reduce the proton gradient across the inner mitochondrial membrane, thereby dissipating energy as heat (7) and thus increasing energy expenditure. Strong evidence indicates that cancer-induced cachexia induces UCP2 and UCP3 at the transcriptional and translational levels in skeletal muscle via tumor necrosis factor alpha (TNFα) (12–14). This induction correlates directly with antioxidative activity as production of reactive oxygen species (ROS) increases as a result of mitochondrial dysfunction (15,16). Inflammatory cytokines are considered to be mediators of and targets for cancer cachexia (17–20); they have been reported to lead to post-transcriptional activation of the peroxisomal proliferator activator receptor γ coactivator 1 α (PGC-1α) via the p38 mitogen-activated protein kinase (MAPK) pathway, resulting in increased respiration in muscle cells (21). In addition, it has been shown that decreased PGC-1α expression leads to profoundly reduced mitochondrial content and activity (22), while increased PGC-1α protein levels have been observed in a rat cancer cachexia model (23). It is now well established that mitochondrial function can be altered via coordinated changes in gene expression (24).

*In vivo* NMR spectroscopy enables quantification of intracellular physiological variables in an organism without removal or destruction of the tissue to be examined, as other techniques usually require to make such an assessment (25,26). Indeed, *in vivo* NMR represents a significant advance in the study of mitochondrial function by providing measurements under physiological conditions in intact skeletal muscle, thus eliminating *in vitro* artifacts. With this methodology, researchers can measure the net skeletal muscle rate of oxidative ATP synthesis catalyzed by mitochondrial ATPase (27,28), which by definition is proportional to oxygen consumption by the P/O ratio (the ratio of the net rate of ATP synthesis by oxidative phosphorylation to the rate of oxygen consumption) (29,30). Furthermore, *in vivo* NMR in combination with whole-genome analysis of gene expression, which provides a snapshot of the transcriptome in a specific tissue in response to experimentally defined physiological conditions, provides a systematic approach for studying complex systems, such as cancer-induced muscle wasting, by integrating mitochondrial function data with information about mitochondrial regulation at the cellular and organism levels.

Although mitochondrial dysfunction, has been shown in experimental models of burn injury to lead to skeletal muscle wasting (31–39), it has never been studied in the context of cancer-induced cachexia. The aim of the present study was to study mitochondrial function in cancer cachexia holistically in a suitable preclinical model. To this end, highly cachectic, fast-growing Lewis lung carcinomas, characterized by poorly differentiated cells with a short doubling time (40), were implanted in mice. This model is advantageous owing to the fast rate of muscle wasting produced and lack of anorexia which produce a clear cachectic state characterized by profound muscle wasting (40–44), mimicking closely the pathophysiology of untreated human cancer cachexia. We hypothesize that mitochondrial dysfunction triggered by an altered gene expression program is a major cause of skeletal muscle wasting in cancer. To test this hypothesis, *in vivo* NMR combined with whole-genome expression analysis was applied to intact Lewis lung carcinoma-inoculated mice and the data were compared to data from control (C) tumor-free mice to assess alterations in the tumor bearing (TB) animals’ bioenergetic status and to characterize concomitant gene expression patterns in cancer-induced cachectic versus control skeletal muscle tissue.

## Materials and methods

### Animals

C57Bl/6 mice (20–25 g) (Charles River Laboratories, Boston, USA) were used as a representative inbred stock and reliable population for the microarray studies. The animals were maintained at 22±2°C with a regular light-dark cycle (lights on from 8:00 am to 8:00 pm) and had free access to standard rodent chow and water. The diet consisted of 54% carbohydrate, 17% protein, and 5% fat (the residue was non-digestible material). Food intake was measured daily; food provided daily was pre-weighted. After 24 h, remaining food was weighted and subtracted from the initially provided food. The net value of food consumed every 24 h gave the rate of food intake. Only male mice were used in order to avoid the variability that can result from the female estrous cycle. All animal experiments were approved by the Subcommittee on Research Animal Care of Massachusetts General Hospital, Boston.

### Tumor implantation

Mice were inoculated with tumor according to an established protocol (43) under short-time isoflurane anesthesia (3% in O_2_) as described previously (43). Animals were randomized into tumor-free control (C) and tumor-bearing (TB). TB-mice received an intramuscular (right hind leg) inoculum of 4×10^5^ Lewis lung carcinoma cells obtained from exponential tumors.

### Evaluation of cancer induced cachexia

Fourteen days after tumor transplantation, the mice were weighed and anesthetized with an intraperitoneal (i.p.) ketamine (87 mg/kg) and xylazine (13 mg/kg) injection. Tumor, tissues of interest and blood were collected. All mice were then administered a lethal dose of pentobarbital (200 mg/kg, i.p.). The cancer-induced cachexia was evaluated by measuring: i) the total body and carcass (muscle + bone + skin) weights, ii) the weight of gastrocnemius, tibialis, soleus and extensor digitorum longus (EDL) muscle weights in the contralateral (left leg) to the tumor bearing leg as described previously (43), and iii) the TNFα, interleukin-6 (IL-6) and interleukin-10 (IL-10) levels in blood using Q-Plex™ Mouse Cytokine/Chemokine kit by Quansys Biosciences laboratory (USA). (www.quansysbio.com/products-services/sample-testing).

### ^31^P NMR spectroscopy

#### Data acquisition

The theoretical basis of saturation transfer experiments has been described previously by Forsen and Hoffman (45). Animals were subjected to *in vivo* ^31^P NMR spectroscopy 14 days after tumor inoculation. The mice were transiently anesthetized with a mixture of isoflurane (3.0%) and O_2_ (2.0 l/min) delivered through a nose cone and placed in a customized restraining tube. Each animal’s left hind limb was placed into a solenoid coil (four turns; length, 2 cm; diameter, 1 cm) tuned to ^31^P frequency (162.1 MHz). During the MR imaging, mice were kept anesthetized with a mixture of isoflurane (1.5%) and O_2_ (0.6 l/min). The rectal body temperature was maintained at 37±1°C using heated water blankets. All *in vivo* ^31^P NMR experiments were performed in a horizontal bore magnet (proton frequency at 400 MHz, 21 cm diameter, Magnex Scientific, Varian, Palo Alto, CA, USA) using a Bruker Advance console. Field homogeneity was adjusted using the ^1^H signal of tissue water. A 90° pulse was optimized for detection of phosphorus spectra (repetition time 2 sec, 400 averages, 4,000 data points). Saturation 90° selective pulse trains (duration, 36.534 ms; bandwidth, 75 Hz) followed by crushing gradients were used to saturate the γATP peak. The same saturation pulse train was also applied downfield of the inorganic phosphate (Pi) resonance, symmetrically to the γATP resonance. T1 relaxation times of Pi and phosphocreatine (PCr) were measured using an inversion recovery pulse sequence in the presence of γATP saturation. An adiabatic pulse (400 scans; sweep width, 10 kHz; 4,000 data points) was used to invert Pi and PCr, with an inversion time between 152 and 7,651 ms.

#### Data analysis

^31^P NMR spectra were analyzed using the MestRe-C NMR software package (Mestrelab Research, NMR solutions, website: www.mestrec.com). Free induction decays were zero-filled to 8,000 points and apodized with exponential multiplication (30 Hz) before Fourier transformation. The spectra were then manually phased and corrected for baseline broad features. The Levenberg-Marquardt algorithm was used to least-square-fit a model of mixed Gaussian/Lorentzian functions to the data. Similarly, the T1obs relaxation time for Pi and PCr was calculated by fitting the function y = A1[1-A2e^-(t/T1obs)^] to the inversion recovery data, where y is the z magnetization and t is the inversion time.

#### Total RNA extraction

The mice were anesthetized by intraperitoneal injection of ketamine (87 mg/kg) and xylazine (13 mg/kg) and the gastrocnemius muscle contralateral (left) to the TB-hind leg was rapidly excised, weighed, and frozen in liquid nitrogen. Left gastrocnemius muscles excised from C animals served as controls specimens. All mice were then administered a lethal dose of pentobarbital (200 mg/kg, i.p.). Frozen biopsies from TB and C mice (n=4) were immersed in 1 ml TRIzol^®^ (Gibco-BRL, Invitrogen, Carlsbad, CA) for RNA extraction. Each muscle specimen was homogenized for 60 sec with a Brinkman Polytron 3000 homogenizer before extraction of total RNA. Chloroform (200 *μ*l) was added to each homogenized muscle specimen and mixed by inverting the tube repeatedly for 15 sec. After centrifugation at 12,000 × g for 15 min, the upper aqueous phase was transferred by pipet to a new tube and precipitated by adding 500 *μ*l of isopropanol. Further centrifugation at 12,000 × g for 10 min condensed the RNA pellet, which was then washed with 500 *μ*l of 70% ethanol and centrifuged at 7,500 × g for 5 min prior to air drying. The pellet was resuspended in 100 *μ*l DEPC-H_2_0. An RNeasy kit (Qiagen, Germantown, MD) was used to purify the RNA according to the manufacturer’s protocol. Purified RNA was quantified by UV absorbance at 260 and 280 nm and stored at −70°C for DNA microarray analysis.

#### Gene array hybridization and analysis

Biotinylated cRNA was generated from 10 *μ*g aliquots of total RNA, and hybridized onto MOE430A oligonucleotide arrays, which were subsequently stained, washed, and scanned. All procedures followed standard Affymetrix protocols (Santa Clara, CA). The hybridized array image data files were converted to cell intensity (CEL) files in Microarray Suite 5.0 (MAS 5.0, Affymetrix). The data were scaled to a target intensity of 500, and Genespring GX (version 7.3) software (Agilent Technologies) was employed for statistical analysis of differential transcript expression using the Welch t-test for multiple testing correction and Benjamini and Hochberg False Discovery Rate (cut-off of 5% false discovery rate and 2-fold change). Probe sets representing the same transcript were ordered on their corresponding unigenes, and the 3′-most probe set was selected from combined lists of all probe sets. A collection of genes with experimental evidence was compiled using MOE430A chip annotation (Affymetrix, retrieved December, 2009) compiled from GeneSpring GX (version 7.3). Statistically significant sets of functionally related genes were selected using overrepresentation statistics calculated as hypergeometric probabilities using all genes selected in each experiment that had Gene Ontology annotation for biological process (46) using Gene Ontology Analysis (GeneSpring GX, version 7.3). Functions’ P-values were estimated using 0.05 as the cut-off point (GeneSpring GX, version 7.3). Functional categories that did not have at least two genes were removed.

## Results

### Lewis lung carcinoma inoculation decreased body, carcass and skeletal muscle weights

Food intake during the experiment did not differ between TB and C groups. Fourteen days after tumor implantation mean total body weight (BW) of TB mice had decreased by 17% relative to pre-inoculation (Table I). BW loss was associated with a 27.3% decrease in carcass weight (muscle + bone + skin) (Table I). Muscle mass was decreased 14 days after tumor implantation in all tissues studied, with the most substantial decreases being observed in the gastrognemnius (50.2% decrease), tibialis (49.0% decrease), and EDL (32.8% decrease) muscles (Table I).

### Lewis lung carcinoma inoculation increased blood levels of cytokines

TB mice had elevated levels of the procachectic cytokines TNFα (77.2% greater than C; P=0.02) and IL-6 (8,120.1% greater than C; P=0.01) 14 days after tumor implantation. Moreover, IL-10, which was undetectable in C mice, was also present at high levels (26.12±4.06 pg/ml) in TB mice.

### Cancer cachexia reduced ATP synthesis rate

^31^P NMR spectra acquired from TB and C mice, before and after saturation of the γATP resonance, with the mean results and their percent change (Δ) presented in Table II. The unidirectional synthesis rate of the P_i_ ➝ γ-ATP reaction in TB mice was 47% lower than that observed in C mice (P=0.029). This synthesis rate involves measurements from NMR and from a biochemical assay (ATP concentration measurement), and both were significantly decreased in the cancer mice. The NMR-measured fractional change ΔM/M_0_ was decreased by 37% in TB mice relative to the C group (percent change in ΔM/M_0_, Table I). ATP concentration (14 days post-inoculation) was lower in TB than in C mice by approximately 27% (Table II), a difference that approached significance (P=0.054) in the unidirectional (one-tailed) t-test. ATP synthesis rate was significantly reduced in TB mice (47% lower than in C) in the unidirectional (one-tailed) t-test (P=0.029). The fractional change, ΔM/M_0_, and the observed spin lattice relaxation time, T_1obs_, were used to calculate the κ_f_ rate constant using the equation (1/T_1obs_) × (ΔM/M_0_). The ATP synthesis flux was then obtained as the product of κ_f_ and P_i_ concentration. Accordingly, the unidirectional synthesis rate of the PCr ➝ γ-ATP reaction was also 27% lower in TB mice compared to C, a difference that was significant according to a unidirectional (one-tailed) t-test (P=0.036).

### Cancer-induced cachexia affected several cellular functions in skeletal muscle

Analysis of transcriptome studies identified 611 genes as differentially expressed in skeletal gastrocnemius muscle in TB versus C mice (P<0.05). Comparison of these 611 differentially expressed genes to annotations in the Gene Ontology Consortium enabled functionally related sets of genes to be identified and subsequent analysis of these data indicated that several cellular functions were altered by cancer-induced cachexia (Fig. 2). The four most prominently affected functions were related to inflammation and immune response, and these effects predominantly involved significant increases in transcription. The four next most prominently affected functional categories involved genes whose products mediate protein metabolism, especially protein degradation. Specifically, as shown in Fig. 2, transcription of molecules involved in protein catabolism, protein depolymerization, and proteasome-related was altered, mostly in the direction of upregulation. The ubiquitin-proteasome pathway in particular was implicated as a major process of protein degradation in cancer-induced cachexia. Transcription of protein maturation related genes was universally down-regulated in TB mice.

The transcriptome analysis revealed cancer-induced alterations in cellular lipid metabolism and lipid catabolism in TB mice relative to C mice (Fig. 2), indicating that cancer-induced cachexia also begets abnormal lipid metabolism. Among the down-regulated genes (data not shown) was stearoyl-Coenzyme A desaturase 1, which encodes for the first (step-limited) enzyme in unsaturated fatty acid biosynthesis. Phospholipase A2 and phospholipase C, which have roles in several lipid metabolic and signaling pathways, were also differentially affected. Genes related to skeletal muscle anti-oxidative capacity were affected by cancer cachexia as well (last two functional catagories listed in Fig. 2). While increased transcription of genes related to oxidative stress response was evident, superoxide metabolism genes were down-regulated.

### Differential expression of key mitochondrial genes in cancer cachexia

Fig. 3 shows the effect of cancer-induced cachexia on the expression levels of selective genes related to mitochondrial function. Specifically, uncoupling protein 3 (UCP3), and forkhead box O 3α (FoXO3α) expression levels were significantly upregulated (P=0.03 and P=0.04 respectively). As well as pyruvate dehydrogenase kinase 4 (PDK4), an inhibitor of pyruvate dehydrogenase complex (P=0.01). Meanwhile, peroxisome proliferator activated receptor-γ coactivator-1ß (PGC-1ß) was significantly down-regulated (P=0.04).

It is noteworthy that the present experiments revealed also altered expression of genes known to be involved in the IGF-1/AKT pathway which regulates: a) signaling pathways responsible for FoXOs post-translational activation; and b) protein synthesis and degradation (Fig. 3). More specifically, our data suggest that cachexic animals had decreased AKT activation since insulin-like growth factor 1 (IGF-1), insulin receptor substrate 1 (IRS-1), and IGF binding protein 5 (IGFBP5) were all significantly down- regulated in TB mice (P=0.03, P=0.02 and P=0.048 versus C, respectively). Furthermore, the ubiquitin-proteasome pathway related genes atrogin-1 and ubiquitin-conjugating enzyme E3 (UBE3α) were upregulated in skeletal muscle of TB mice (P=0.0495 and P=0.0252 versus C, respectively); and the eukaryotic translation initiation factor 4E binding protein 1 (EIF4Ebp1) was also significantly up-regulated in TB mice (P=0.0077 versus C). Genes related to antioxidative capacity were also differentially expressed in TB mice: superoxide dismutase 2 (SOD2) was down-regulated (P=0.0111 versus C) while glutathione peroxidase 3 (GPX) was upregulated (P=0.0068 versus C).

## Discussion

In this study, we used a combined approach of *in vivo* NMR together with whole-genome expression analysis to investigate mitochondrial function and elucidate a possible regulatory molecular mechanism that may mediate skeletal muscle wasting in an experimental preclinical murine model of cancer cachexia. Our animal model was validated by our findings of weight decreases in total body, carcass and several skeletal muscles 14 days after tumor implantation, observations that corroborated prior findings (43). Further validation of our model was provided by our genomic data which showed aberrant gene expression in several cellular functions related to cancer cachexia; the affected genes were most prominently found to be involved in immune-inflammatory response, protein degradation, lipid metabolism and antioxidative defense mechanisms. Increased immune-inflammatory response is consistent with the pathophysiology of cancer-induced cachexia described in previous reports (47,48), while protein degradation is considered to be a major cause of cachexia (2). Furthermore, the cancer-induced alterations in cellular lipid metabolism reported in this study are also indicative of abnormal lipid metabolism in cachexia, in agreement with previous observations (49).

The present *in vivo* NMR spectroscopy studies, which allowed measurements of physiological biomarkers (25,26), showed a significantly reduced ATP synthesis rate in cachexic mice suggestive of bioenergetic mitochondrial dysfunction. Our accompanying whole-genome expression experiments complemented our NMR findings, revealing aberrant expression of genes involved in mitochondrial biogenesis (PGC-1ß), and uncoupling (UCP3) in a clinically relevant cancer cachexia model. Interestingly, our NMR findings of mitochondrial dysfunction in the skeletal muscle of animals exhibiting cancer cachexia are similar to prior observations in murine models of burn trauma (31,37). *In vivo* ^31^P-NMR saturation-transfer can non-invasively measure fast enzyme reaction exchange rates (50) and, in particular, the net rate of oxidative ATP synthesis catalyzed by mitochondrial ATPase in skeletal muscle, which is proportional to the oxygen consumption rate according to the P:O ratio (the ratio of the net rate of ATP synthesis by oxidative phosphorylation to the rate of oxygen consumption) (29,30). It has been proposed that NMR-measured unidirectional ATP synthesis flux primarily reflects flux through F_1_F_0_-ATP synthase, with negligible influence of the coupled glyceraldehyde-3-phosphate dehydrogenase and phosphoglycerate kinase reactions for ATP production (28,51). Because these enzymes occur at near equilibrium, unidirectional production of ATP can be high. Furthermore, since cancer cachexia upregulates expression of PDK4 (in mouse Fig. 3; in rat, see (23), a well established inhibitor of the pyruvate dehydrogenase complex which is involved in controlling the use of glucose-linked substrates as sources of oxidative energy via glycolysis (52), we can assume that the contribution of glycolytic reactions to unidirectional ATP synthesis flux is negligible.

In agreement with our NMR findings, our genomic experiments revealed reduced expression of PGC-1ß, a mitochondrial biogenesis transcription factor. Reduced expression of PGC-1ß has also been observed in murine burn-induced skeletal muscle wasting models (31,37), while increased PGC-1α protein levels have been previously reported in a rat cancer cachexia model (23), strongly supporting the view that PGC-1s play a key role in cancer-induced muscle wasting. Specifically, it has been suggested that PGC-1α protects skeletal muscle from atrophy (53) while PGC-1ß expression has been associated with an increase in ATP-consuming reactions (24). Moreover, reduced PGC-1 expression levels have also been correlated to profoundly reduced mitochondrial content and activity (22); these effects may be due to the action of UCPs (34) given that down-regulation of PGC-1s is accompanied by increased UCP expression in murine models of both cancer- (12,14,23) and burn-related cachexia (31,54). Increased levels of UCPs dissipate the proton gradient and lower the mitochondrial membrane potential, a process known to increase energy expenditure by dissipating energy as heat (7). The presently observed upregulation of UCP3 in cachexic animals corroborates previous findings (12–14,16).

Indeed, this increase in UCP3 gene expression suggests that there was mitochondrial uncoupling in TB mice since increased levels of UCPs lower the mitochondrial membrane potential (55) and thus greatly reduce the amount of ROS in mitochondria (56,57), a major site of ROS production in the cell (58,59), especially in conditions characterized by high TNFα levels (60), such as cancer. To this end, it has been proposed that upregulation of UCPs in cancer may be directly related to the antioxidative capacity of skeletal muscle, thus involving UCPs in a counter-regulatory mechanism to lower ROS production; this process is thought to involve the muscle anabolic cytokine IL-15 (16). UCP expression has been reported to be upregulated by procachectic cytokines (TNFα and IL-6) via p38MAPK-dependent post-translational activation of PGC-1s (21). Our data agree with this notion, as we observed increased levels of the procachectic cytokines TNFα and IL-6 in TB mice relative to C mice (by 77.2% and 8,120.1%, respectively).

A principal finding of the present study was that down-regulation of PGC-1ß in TB mice was accompanied by a down-regulation of SOD2 as well as an upregulation of GPX. Interestingly, it has previously been shown that PGC-1s increase gene expression of SOD2, GPX and enzymes responsible for glutathione biosynthesis (24), and both SOD2 and GPX have been implicated in cancer-induced oxidative stress response (61–63). Our results indicate that muscle defense against oxidative stress in cancer is mediated largely by GPX, corroborating the observed increase in genes ascribed to the ‘response to oxidative stress’ function. Meanwhile, genes ascribed superoxide metabolism function were expressed at significantly lower levels in TB mice than in C mice, indicating that the defense mechanisms functioning in cancer cachexia are not adequate to handle the oxidative stress with which they are faced.

Another principal finding of our study is that decreased expression of PGC-1ß was accompanied by increased FoXO3α gene expression levels. This finding is consistent with a previous report suggesting that PGC-1s inhibit FoXOs-dependent transcription (53) and provides support for the view that PGC-1ß may play a central regulatory role in cancer-induced cachexia given that FoXOs are involved in multiple signaling pathways and play critical roles in numerous physiological and pathological processes including cancer (64). Specifically, FoXOs are under regulatory influences at multiple levels, including phosphorylation, acetylation/deacetylation, ubiquitination, and protein-protein interactions (64). Furthermore, in the present study, we also report aberrant expression of genes known to be involved in the IGF-1/AKT pathway, which has been reported to play a role in FoxOs post-translational activation (65,66). Involvement of the IGF-1/AKT pathway in cancer cachexia (67) was confirmed by our data showing decreased AKT activation as evidenced by down-regulated IGF-1, IRS-1 and IGFBP5. Consequently, decreased AKT activation could permit dephosphorylation of FoXOs and thus translocation of FoXOs to the nucleus, where they are activated and promote upregulation of genes related to protein degradation (53,66,68,69), namely atrogin-1 and ubiquitin-conjugating enzyme E3, thus leading to cachexia. Genes related to the structure of the proteolytic system (proteasome function in Fig. 2) were also upregulated in TB mice, further implicating activation of the ubiquitin-proteasome pathway as a major generator of protein degradation in cancer-induced cachexia, as previously reported in cancer patients exhibiting weight loss (70).

The hypothesis that IGF-1/AKT attenuation contributes to cancer cachexia is supported by the presently observed elevated levels of the EIF4Ebp1 in TB mice, as EIF4Ebp1 levels have been found to correlate with cancer development and cachexia (71,72) due to inhibition of protein synthesis (73) via binding to eukaryotic initiation factor 4E (eIF4E) (74). Specifically, hypophosphorylated EIF4Ebp1 binds to eIF4E and prevents recruitment of the 40S ribosomal subunit to mRNA, thus inhibiting cap-dependent translation. Binding of EIF4Ebp1 to eIF4E is regulated by mTOR-mediated phosphorylation (73). Upon its activation, mTOR phosphorylates EIF4Ebp1; hyperphosphorylated EIF4Ebp1 is released from eIF4E, leading to an increase in cap-dependent translation (75). Given that mTOR activation is regulated mainly by the IGF-1/AKT pathway, attenuation of AKT activity as observed in our experimental cancer cachexia model should further facilitate protein translation inhibition due to mTOR inactivation and the consequent hypophosphorylation of EIF4Ebp1.

**Figure 1:**
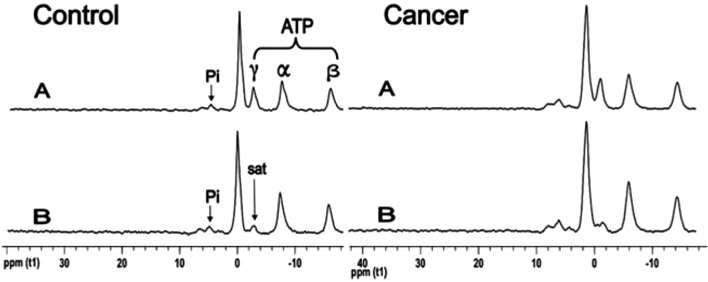
NMR spectra of *in vivo* ^31^P NMR saturation-transfer performed on the hind limb skeletal muscle of awake mice. Representative summed ^31^P-NMR spectra acquired from C and TB mice before (A) and after (B) saturation of the γ-ATP resonance. The arrow on γ-ATP indicates the position of saturation (sat) by rf irradiation (−2.4 ppm). ppm, chemical shift in parts per million.

**Figure 2:**
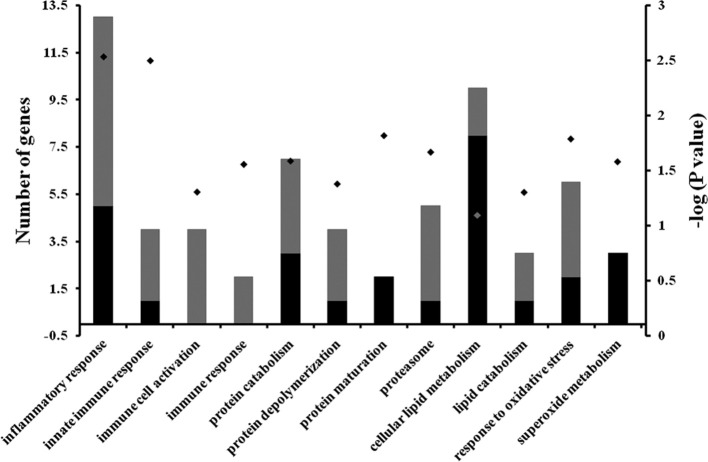
Distribution of genes differentially expressed in gastrocnemius muscle of TB animals relative to C animals among 14 functional categories showed in the X axis, as identified by using Gene Ontology and KEGG metabolic pathways at P≤0.05. Gray bars indicate the number of upregulated genes while black bars correspond to down-regulated genes in the gastrocnemious muscle of TB animals versus C animals (left Y axis). The negative log10 of P-values represented by diamonds are indicated in the right Y axis.

**Figure 3:**
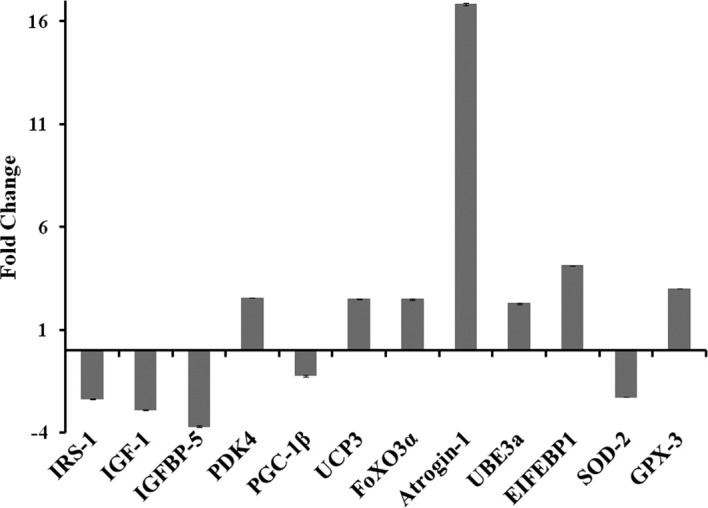
Summary of the magnitudes of change in mRNA expression of mitochondrial function-related and other cancer-induced cachexia-related genes 14 days after cancer transplantation. IRS-1, insulin receptor substrate 1; IGF-1, insulin-like growth factor 1; IGFBP-5, insulin-like growth factor binding protein 5; PDK4, pyruvate dehydrogenase kinase 4; PGC-1ß, peroxisome proliferator-activated receptor-γ coactivator-1ß; UCP3, uncoupling protein 3; FoxO3α, Forkhead box O3α; ATROGIN-1, F-box only protein 32; UBE3a, ubiquitin protein ligase E3A; SOD-2, superoxide dismutase 2; GPX, glutathione peroxidase 3.

**Figure 4:**
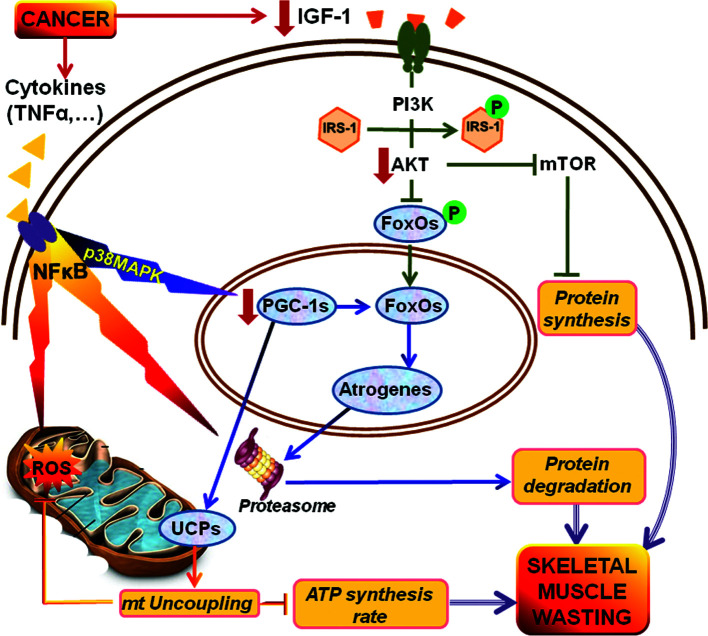
Putative PGC-1-mediated mechanism of cancer-induced skeletal muscle wasting. In this model, significant down-regulation of PGC-1ß expression causes dysregulation of mitochondria, resulting in reduced ATP synthesis and increased ROS production, most likely due to PGC-1-dependent upregulation of UCPs. Meanwhile, reduced PGC-1ß gene expression levels result in a failure to down-regulate expression of FoXO3α, which normally suppresses atrogen expression and proteolysis. Insulin-stimulated IGF-1/AKT pathway signaling is also significantly attenuated. Thus, AKT does not phosphorylate FoXOs and, consequently, favors further upregulation of atrogenes, thus leading to skeletal muscle wasting. Moreover, reduced IGF-1/AKT signaling further facilitates protein translation inhibition due to mTOR inactivation which also results in cachexia. Meanwhile, PGC-1ß may affect IGF-1 and IRS-1 expression thereby producing insulin resistance in skeletal muscle. IRS-1, insulin receptor substrate 1; IGF-1, insulin-like growth factor 1; PGC-1s, peroxisome proliferator-activated receptor-γ coactivators-1; UCPs, uncoupling proteins; FoXOs, forkhead box O proteins; ROS, reactive oxygen species; TNFα, tumor necrosis factor α; NF-κB, nuclear factor-κB.

In light of the afore-mentioned lines of evidence and previous reports, we propose here that PGC-1 acts as a key regulator of cancer-induced bioenergetic dysfunction and muscle wasting in experimental cancer cachexia, as illustrated in Fig. 4. Specifically, we propose that cancer induction of cachexia involves: i) decreased expression of PGC-1ß, which normally inhibits FoXOs; and ii) attenuated AKT activity, which both enable activation of FoXO3α and upregulate the expression of atrogenes, which then facilitate muscle degradation. In addition, attenuation of AKT can inhibit protein synthesis since the IGF-1/AKT pathway normally contributes to protein synthesis via mTOR. Meanwhile, decreased expression of PGC-1ß appears to: a) upregulate UCP3, leading to uncoupling and reduced ATP synthesis rate; and b) alter immuno-inflammatory gene expression in skeletal muscle [in cachexia Fig. 1; in experimental burn injury (31,33,34,36)]. This supposition is in agreement with other studies suggesting that upregulation of mRNA and protein expression of UCP2 and UCP3 (12–14) correlates directly with antioxidative capacity (15,16) in response to elevated TNFα-induced ROS production in cancer (60).

In conclusion, because hallmarks of skeletal muscle wasting can be detected by *in vivo* NMR, our findings are clinically relevant in as much as they can be used as molecular medicine biomarkers that can be translated to humans. Moreover, if PGC-1s mediate key regulatory functions in cancer-induced cachexia as suggested by the present findings, they could serve as funnels where several oncogenic/cachectic signals (cytokines, ROS, IGF-1) converge to drive the downstream physiological response (mitochondrial dysfunction, antioxidative and immune-inflammatory response, protein synthesis and degradation) of skeletal muscle to malignancy. Because PGC-1ß is a highly inducible factor in most tissues that responds to common calcium and cAMP signaling pathways, it is conceivable that drugs could be developed with the ability to induce PGC-1ß and thereby directly alleviate mitochondrial dysfunction. Such a novel therapeutic approach could revolutionize cancer cachexia treatment.

## Figures and Tables

**Table I:** Cancer cachexia effects in C57BL/6 mice.

	Control	Tumor-bearing (day 14)	P-value
Initial BW (g)	17±0.2 (n=5)	17±0.5 (n=8)	NS
Final BW (g)	22±1 (n=5)	14±1 (n=8)	<0.001
% weight change	29%	−17%	
Carcass (mg/100 g initial BW)	77±2 (n=5)	56±1 (n=8)	<0.001
Muscles weights (mg/100 g initial BW)			
Gastrocnemius	764±16 (n=6)	381±6 (n=8), −50.2%	<0.001
Tibialis	241±8 (n=6)	123±7 (n=8), −49%	<0.001
Soleus	42±1 (n=6)	37±3 (n=8), −11.9%	NS
EDL	58±4 (n=6)	39±3 (n=8), −32.8%	<0.01

Results are expressed as mean ± SEM for the number of animals indicated in parentheses. EDL, extensor digitorum longus; NS, not significant; carcass weight, muscle + bone + skin.

**Table II:** Results of *in vivo* ^31^P-NMR saturation transfer experiments performed on the hindlimb skeletal muscle of mice.

	Control (n=10)	Tumor-bearing (n=6)	Δ(%)	P-value
ATP synthesis flux (reaction Pi➝γATP)				
ΔM/M_0_	0.484±0.036	0.304±0.051	−37.2	0.011
T_1obs_ (s)	1.59±0.14	1.50±0.14	−5.6	NS
κ_f_ (s^−1^)	0.304±0.026	0.203±0.038	−33.2	0.038
ATP (mmol/g)	1.19±0.14	0.87±0.19	−26.9	0.054
P_i_ (mmol/g)	0.280±0.062	0.222±0.079	−20.7	NS
ATP synthesis rate (mmol/g/s)	0.085±0.013	0.045±0.013	−47.1	0.029
ATP synthesis flux (reaction PCr➝ATP)				
ΔM/M_0_	0.24±0.02	0.26±0.02	+8.3	(NS)
T_1obs_ (s)	1.59±0.20	1.50±0.27	−5.7	(NS)
κ_f_ (s^−1^)	0.15±0.02	0.17±0.03	+13.3	(NS)
ATP (*μ*mol/g)	1.19±0.28	0.87±0.19	−26.9	(NS)
PCr (*μ*mol/g)	4.13±0.99	2.65±0.59	−35.8	0.015
ATP synthesis rate (*μ*mol/g/s)	0.62±0.17	0.45±0.13	−27.4	0.036

Values are means ± SEM; ΔM/M_0_ is the fractional change in P_i_ or PCr magnetization as a result of saturation transfer; T_1obs_ is the observed spin lattice relaxation time of P_i_ or PCr during γATP saturation in seconds; κ_f_ is the rate constant for the reactions P_i_ ➝ ATP and PCr ➝ ATP, calculated as (1/T_1obs_) × (ΔM/M_0_). ATP synthesis is calculated as [P_i_] or [PCr] × κ_f_. A bioluminescence assay kit was used to assess ATP concentration. Δ (%), percent change. NS, not significant. Unpaired Student’s t-test was used for the comparisons.
